# Comprehensive design of omnidirectional high-performance perovskite solar cells

**DOI:** 10.1038/srep29705

**Published:** 2016-07-13

**Authors:** Yutao Zhang, Yimin Xuan

**Affiliations:** 1 School of Energy and Power Engineering, Nanjing University of Science & Technology, Nanjing 210094, China; 2 School of Energy and Power Engineering, Nanjing University of Aeronautics & Astronautics, Nanjing 210094, China

## Abstract

The comprehensive design approach is established with coupled optical-electrical simulation for perovskite-based solar cell, which emerged as one of the most promising competitors to silicon solar cell for its low-cost fabrication and high PCE. The selection of structured surface, effect of geometry parameters, incident angle-dependence and polarization-sensitivity are considered in the simulation. The optical modeling is performed via the finite-difference time-domain method whilst the electrical properties are obtained by solving the coupled nonlinear equations of Poisson, continuity, and drift-diffusion equations. The optical and electrical performances of five different structured surfaces are compared to select a best structured surface for perovskite solar cell. The effects of the geometry parameters on the optical and electrical properties of the perovskite cell are analyzed. The results indicate that the light harvesting is obviously enhanced by the structured surface. The electrical performance can be remarkably improved due to the enhanced light harvesting of the designed best structured surface. The angle-dependence for s- and p-polarizations is investigated. The structured surface exhibits omnidirectional behavior and favorable polarization-insensitive feature within a wide incident angle range. Such a comprehensive design approach can highlight the potential of perovskite cell for power conversion in the full daylight.

With the rapid consumption of fossil fuels like coal, oil and natural gas, it is urgent to develop alternative approach to supply sustainable energy production with the growing global energy demand^1^. Solar energy is one of the most potential abundant, clean and renewable energy resources to resolve the energy crisis^2^. Photovoltaic solar cell has been a more and more effective approach to utilize the solar energy. Plenty of efforts have been devoted to the investigation of the solar cell to increase the power conversion efficiency (PCE) and reduce the cost of the solar cell^3^,^4^,^5^,^6^. Solid-state organo-metal halide perovskite solar cells emerged as a kind of promising alternative to the existing photovoltaic technologies^7^. The solid-state perovskite solar cells are derived from the liquid-state dye-sensitized solar cells^8^. Recently, the energy conversion efficiency of the solid-state perovskite solar cells has quickly surpassed that of the conventional dye-sensitized solar cells and attracted more and more attentions^9^.

Perovskite refers to the crystal structure of the mineral family of ABX_3_, where A is the monovalent or divalent metal and B is the tetravalent or divalent metal. X is the chalcogen or halogen^10^. There are hundreds of materials adopt to this crystal structure, such as CsPbCl_3_, BaTiO_3_, (NH4)SnI_3_, and CH_3_NH_3_PbI_3_^11^,^12^,^13^. Because of the excellent solar-to-electricity energy conversion performance of CH_3_NH_3_PbI_3_ material, it is applied to the photovoltaic cell and has attracted great attentions. For a long time, CH_3_NH_3_PbI_3_ was implemented as sensitizer particles in dye-sensitized solar cells (DSSC) with liquid-electrolyte^14^. The function of the electrolyte is to regenerate the oxidized dye and to transport the holes to the external circuit. As a result of the concerns over solvent leakage and corrosion, recent attentions have been focused on replacing this electrolyte with solid-state hole transporter alternatives to create fully solid-state electronic DSSCs^15^. Bach *et al*. developed the perovskite cell by replacing the liquid-electrolyte with solid-state hole-transport material (HTM) 2,2′,7,7′ tetrakis (N,N-di-p-methoxyphenyl-amine) 9,9′ –spirobifluorene (OMeTAD) and the cell performance is obviously improved^16^. In 2012, a strong efficiency improvement to 9% was achieved by Kim *et al*. by replacing the liquid-electrolyte with solid HTM^17^. However, the energy conversion efficiency is still very low, even lower than that of original electrochemical cell. In order to increase the conversion efficiency, great efforts have been devoted to the investigations of the optical and electrical characteristics of solid-state perovskite solar cell. Lee *et al*. replaced the mesoporous n-type TiO_2_ with insulating Al_2_O_3_ scaffold and found that the great increase in *V*_oc_ is observed and a PCE of 10.9% is achieved^18^. Kim *et al*. reported a highly efficient solar cell based on a submicrometer rutile TiO_2_ nanorod sensitized with CH_3_NH_3_PbI_3_ nanodots^19^. It was found that the short-circuit photocurrent density of 15.6 mA/cm^2^, open-circuit voltage (*V*_oc_) of 955 mV and fill factor (*FF*) of 0.63, which lead to a PCE of 9.4%, are realized by such a perovskite solar cell. Concurrently, the development in the understanding of operation mechanism confirming long distance charge transport has supported the utilization of conventional thin film architectures, which simplifies the fabrication of solution-based perovskite solar cell at low temperature^20^,^21^,^22^. According to the Snaith’s investigation, the intrinsic CH_3_NH_3_PbI_3_ layer was sandwiched by the p-type HTM and n-type TiO_2_ layer. Consequently the whole structure appears as p-i-n architecture^23^. The energy conversion efficiency was greatly increased by the improvements in the material processing and solar cell fabrication. Liu and Kelly used a thin film of ZnO particles as electron-transport material in CH_3_NH_3_PbI_3_ based solar cells. The PCE was as high as 15.7% and the processing temperature was greatly reduced^24^. Zhou *et al*. remarkably suppressed the carrier recombination in the absorber, facilitated carrier injection into the carrier transport layers, and maintained good carrier extraction at the electrodes by controlling the formation of the perovskite layer and careful choices of other materials^25^. The energy conversion efficiency was observed to be boosted to 16.6% on average with the highest efficiency of 19.3% in a planar geometry without antireflective coating. With an unprecedented short development period of perovskite solar cells, the PCE has reached over 20%^26^.

The prior investigation of the perovskite solar cell was prominently performed by the experimental methods and the electrical property is merely considered. Recently, several groups have reported their theoretical research about perovskite solar cell performance, including optical and electrical features. Liu *et al*. simulated the planar heterojunction-based perovskite solar cells with AMPS-1D^27^. The simulation results revealed a great dependence of energy conversion efficiency on the thickness and defect density of the perovskite layer. The energy conversion efficiency over 20% was obtained. However, the optical properties were not included in their research and the incident solar energy was assumed to be completely absorbed. Ball *et al*. used an optical model of perovskite planar heterojunction solar cells which has been optimized by the transfer-matrix formalism using experimentally determined complex refractive index to analysis the optical and electrical cell performances^28^. The simulated and experimental results coincided well with each other. The short-circuit current (*J*_sc_) density of 22.6 mA/cm^2^ is obtained under AM1.5 sunlight with 104.7 mW/cm^2^ irradiance. Yet up to now, there is little investigation concerning the effect of micro-structure on the optical and electrical performances of perovskite solar cells.

Due to the changing incident angle of sunlight, the auto-solar tracker is generally employed to follow the sun. However, the complicated auto-solar tracker is expensive and delicate as a result of the existence of moving components. Hence it is necessary and more attractive to investigate the omnidirectional structured surfaces to achieve light trapping in solar cells. For example, the reflection is suppressed by sub-wavelength Mie resonators within a broad wavelength band and wide incident angles^29^. Moth-eye structure has been proved to possess excellent broadband omnidirectional optical features. Song *et al*. have proved that the moth-eye structure could be used to realize the omnidirectional light trapping in GaAs solar cells^30^. Tommila *et al*. fabricated the SiN_x_ coated moth-eye coating by nanoimprint lithography directly on dilute nitride solar cell^31^. The mean reflection within the spectral range of 320–1800 nm remained under 5% for incident angles up to 45° and the clear increase of electrical performance was verified. Yamada *et al*. characterized the antireflection moth-eye films placed on top of crystalline silicon photovoltaic modules by indoor and outdoor experiments in conversion efficiency^32^. According to their investigations, it is found that the use of antireflection moth-eye films offers the best advantages. With the large aspect ratio of the height to the width, the surface possessed angle-independent spectral feature^33^. In addition, the sunlight is the natural light, which includes all polarization states. The light-trapping is remarkably influenced by the polarization of the incident light at oblique incidence. Therefore, the structured surface of the perovskite cell is expected to possess high absorption for all polarizations.

Yet up to now, the investigation of perovskite solar cell is mainly focused on the experimental research and the film stack is the mainstream structured surface, in which the performance of the perovskite solar cell is generally dependent on the incident angle. The systematical design method for the optical-electrical properties of the perovskite solar cell is scarcely developed. The absorption of the cell is not high enough and the micro-structured surface to enhance the light harvesting is little considered. The incident angle-dependence and polarization-sensitivity are hardly involved.

This paper describes a comprehensive design approach with optical-electrical simulation for perovskite solar cells. The selection of structured surface, effects of geometry parameters, incident angle-dependence and polarization-sensitivity are involved in the simulation. The optical absorption is calculated by the finite-difference time-domain (FDTD) method and the electrical performance is obtained by solving the coupled nonlinear equations of Poisson, continuity, and drift-diffusion equations. Experimental results in the literature are used to validate the theoretical model and the parameters employed in the simulations. The optical absorption and electrical properties of five different structured surfaces are calculated and compared to select a best structured surface with the highest PCE for perovskite cell. The effects of the geometry parameters on the optical response and electrical properties of the structured surface are analyzed. The incident angle-dependence for s- and p-polarizations is investigated.

## Results

### Theoretical model validation

The perovskite cell is generally consisted of SiO_2_, FTO, TiO_2_, CH_3_NH_3_PbI_3_ and HTM. FTO is the cathode and Ag is the anode. TiO_2_ and HTM respectively serve as electron transport material and hole transport material. CH_3_NH_3_PbI_3_ film is the intrinsic photoactive layer. The spiro-OMeTAD is applied as the HTM. The optical constants of SiO_2_, FTO, TiO_2_, CH_3_NH_3_PbI_3_ and HTM used in this paper are obtained from Ball’s investigation, which are measured by the spectroscopic ellipsometry^34^, and the optical constant of Ag is selected from ref. 35. The electrical properties of the materials utilized in perovskite solar cell are listed in Supplementary Table S1. The Shockley-Read-Hall (SRH) recombination and surface recombination are considered in the simulation.

**Figure 1:**
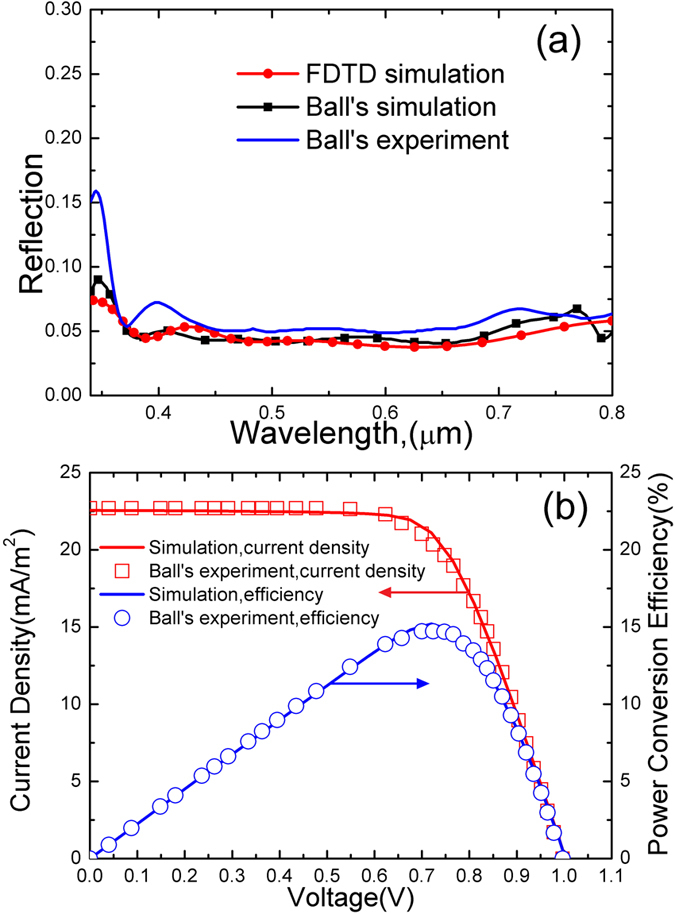
Validation of theoretical model. (**a**) Comparison of the simulated optical properties of planar film structure with the experimental data^28^. (**b**) Comparison of the simulated electrical performances of planar film structure with the experimental data^28^.

The theoretical model is validated against the investigation reported by Ball *et al*.^28^. The spectral features of the structured surface in ref. 28 and the optical generation are calculated by FDTD method. With this optical generation, the current-voltage characteristics are obtained by solving three basic semiconductor equations (see the Methods section for more details). As shown in Fig. 1a, the simulated optical results are compared with the numerical and experimental data from Ball *et al*.^28^. The reflection is about 0.05, which is comparable to that of the AR-coated Si solar cells^36^,^37^. The calculated results coincide well with the theoretical and experimental data from Ball *et al*. The slight deviation between the simulated results and the Ball’s theoretical data, which are obtained by the transfer matrix method, is attributed to the difference between the calculation methods. The simulated electrical characteristics are compared with the experimental data from Ball *et al*. in Fig. 1b. The *J*_sc_ of 22.6 mA/cm^2^ and *V*_oc_ of 1 V are realized. It is found that the numerical results and experimental data are in good agreement across the voltage range from zero to the *V*_oc_ (0–1 V). This implies that the optical and electrical parameters applied here are reasonable. The theoretical method and the established model are valid and applicable.

### Selection of structured surface for perovskite solar cell

**Figure 2:**
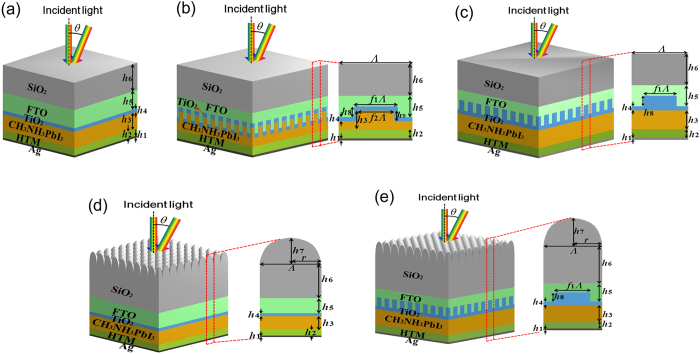
Device architectures and geometry parameter indications of five different structured surfaces. (**a**) Structured surface A: film stack. (**b**) Structured surface B: structured surface with CH_3_NH_3_PbI_3_ grating. (**c**) Structured surface C: structured surface with TiO_2_ grating. (**d**) Structured surface D: structured surface with SiO_2_ grating. (**d**) Structured surface D: structured surface with TiO_2_ grating and SiO_2_ grating. The black dash line indicates the normal direction of the top interface.

In order to select a best structured surface for perovskite solar cell, five possible structured surface, including film stack, structured surface with CH_3_NH_3_PbI_3_ grating, structured surface with TiO_2_ grating, structured surface with SiO_2_ grating and structured surface with TiO_2_ grating and SiO_2_ grating, are considered to enhance light harvesting and PCE herein, as shown in Fig. 2a–e. For convenience, the five structured surfaces are referred to simply as structured surface A, B, C, D and E in order, respectively. The film stack structure in Fig. 2a is the simplest and the most investigated structure of perovskite solar cell. Therefore, the performance of structured surface A is treated as the reference to other structured surfaces. The structured surface is periodically arranged and Λ is the period of structured surface. *r* = Λ/2 is the bottom radius of the SiO_2_ moth-eye grating. *h*_1_ is the thickness of Ag film and *h*_2_ is the thickness of HTM film. *h*_3_ is the thickness of CH_3_NH_3_PbI_3_ structure. *h*_4_ is the thickness of TiO_2_ film and *h*_5_ is the thickness of FTO film. *h*_6_ is the thickness of the SiO_2_ substrate and *h*_7_ is the height of the Si moth-eye structure. *h*_8_ is the height of the TiO_2_ grating. *h*_9_ is the height of the CH_3_NH_3_PbI_3_ grating. *f*_1_ is the filling ratio of TiO_2_ grating. *f*_2_ is the filling ratio of CH_3_NH_3_PbI_3_ grating. These two filling ratios are always set to 0.5 and 0.45, respectively. The period of CH_3_NH_3_PbI_3_ grating is set to equal to the period of moth-eye structure. *θ* is the incident angle. The black dash line indicates the normal direction of the top interface. This paper focuses on the theoretical comprehensive design method of perovskite solar cells and the experiment is not considered. The existing literatures can verify the feasibility of the fabrications of designed structured surfaces. The FTO grating nanostructure is patterned first and the other layers or nanostructures are grown one by one. The SiO_2_ moth-eye grating is patterned on the bottom of SiO_2_ substrate at last. The detail fabrication methods can be found in many published literatures^38^,^39^.

**Figure 3:**
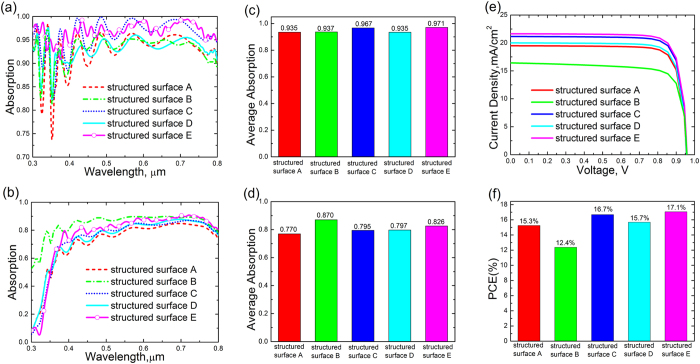
Spectral absorption and electrical properties of five different structured surfaces of perovskite solar cells. (**a**) Overall absorption of five different structured surfaces. (**b**) Intrinsic absorption of five different structured surfaces. (**c**) Average overall absorption of five different structured surfaces. (**d**) Average intrinsic absorption of five different structured surfaces. (**e**) JV-characteristics of five different structured surfaces. (**f**) Power conversion efficiencies of five different structured surfaces.

Since the scattering is strongest when the structural size is comparable to the incident wavelength and the center wavelength of the solar spectrum is around 0.6 μm, the optical absorption of the structured surface with the period of 0.6 μm is highest (Supplementary Fig. S1). Thus the period of the structured surface discussed in this paper is always set to 0.6 μm. The geometry parameters of five structured surfaces are listed in Table 1. The optical absorption and JV-charateristics of different structured surfaces are calculated and the results are compared in Fig. 3. The optical absorption is obviously enhanced by the structured surface, as shown in Fig. 3a. The overall absorption means the absorption of the whole structured surface. It can be found that the structured surface E and structured surface D possess the overall absorption significantly higher than that of the structured surface A due to the influence of SiO_2_ grating. The SiO_2_ grating on one hand provides a smoother gradient effective refractive index; on the other hand, the SiO_2_ grating induces scattering, which further suppresses the reflection. Therefore, the reflection at the SiO_2_/air interface is obviously suppressed and the absorption is enhanced. Simultaneously, it can be observed that the overall absorption of the structured surface E is highest among five structured surfaces due to the double scatterings of the SiO_2_ grating and TiO_2_ grating. The light entering the solar cell is further scattered by the TiO_2_ grating and the absorption is enhanced. For perovskite solar cell, the absorption of intrinsic CH_3_NH_3_PbI_3_ layer is vital because only the photons absorbed by the intrinsic layer can be converted into electricity power. Hence it is of great interest to investigate the intrinsic absorption of the cell. As shown in Fig. 3b, the intrinsic absorption of five structured surfaces is compared to choose a best structured surface with highest intrinsic absorption. It is seen that the intrinsic absorption of structured surface B is remarkably higher than that of other structured surfaces. This is attributed to that the CH_3_NH_3_PbI_3_ grating scatters the light and more photons are absorbed by the CH_3_NH_3_PbI_3_ layer. The intrinsic absorption of the structured surface E is lower than that of the structured surface B while it is higher than that of the rest three structured surfaces. This is mainly resulted from the double scatterings. Figure 3c,d illustrate the average overall absorption and average intrinsic absorption of five structured surfaces. It can be found that the average overall absorption of structured surface E, which is as high as 0.971, is highest among five structured surfaces. The average overall absorption of the planar film structure is lowest. The average intrinsic absorption of the structured surface B reaching as high as 0.870 is highest among five structured surfaces because of the scattering of the CH_3_NH_3_PbI_3_ grating. In addition, although the average intrinsic absorption of the structured surface E is not as high as that of the structured surface B, it still reaches 0.826, which is obviously higher than that of structured surface A. This demonstrates that the absorption of perovskite solar cell can be effectively enhanced by the structured surfaces. For a solar cell, the electrical performance is vitally important and the high PCE is the ultimate destination. The JV-characteristics of five structured surfaces are illustrated in Fig. 3e. It is found that the structured surface E possesses the greatest *J*_sc_, which reaches as high as 21.64 mA/cm^2^, due to the enhanced intrinsic absorption. The *J*_sc_ of the structured surface C is also obviously higher than that of structured surface A and is slightly smaller than that of the structured surface E. This demonstrates that the TiO_2_ material is beneficial for the electron extraction and transport and it is suitable for the electron transport material. Despite of most significant intrinsic absorption among five different structured surfaces, the *J*_sc_ of the structured surface B is remarkably reduced to 16.43 mA/cm^2^. This is attributed to that the surface recombination velocity at CH_3_NH_3_PbI_3_/TiO_2_ interface is significant and the CH_3_NH_3_PbI_3_ grating increases the surface area so that the overall surface recombination is increased. Based on the JV-characteristics in Fig. 3e, the PCEs of five different structured surfaces for perovskite solar cell are obtained and shown in Fig. 3f. The structured surface E has the highest PCE of 17.1%, which is 1.8% higher than the PCE of 15.3% of structured surface A. The PCE of the structured surface B is as low as 12.4% due to the great surface recombination. Thus the structured surface E is the best structured surface among five structured surfaces because of its advanced light-harvesting and solar-to-electricity performances. In the following sections, the discussion is centered on the structured surface E. The height of CH_3_NH_3_PbI_3_ grating *h*_9_ is set to zero. The rest sizes of the structured surface E maintain the same as listed in Table 1.

### Effect of the SiO_2_ grating height *h*_7_

**Figure 4:**
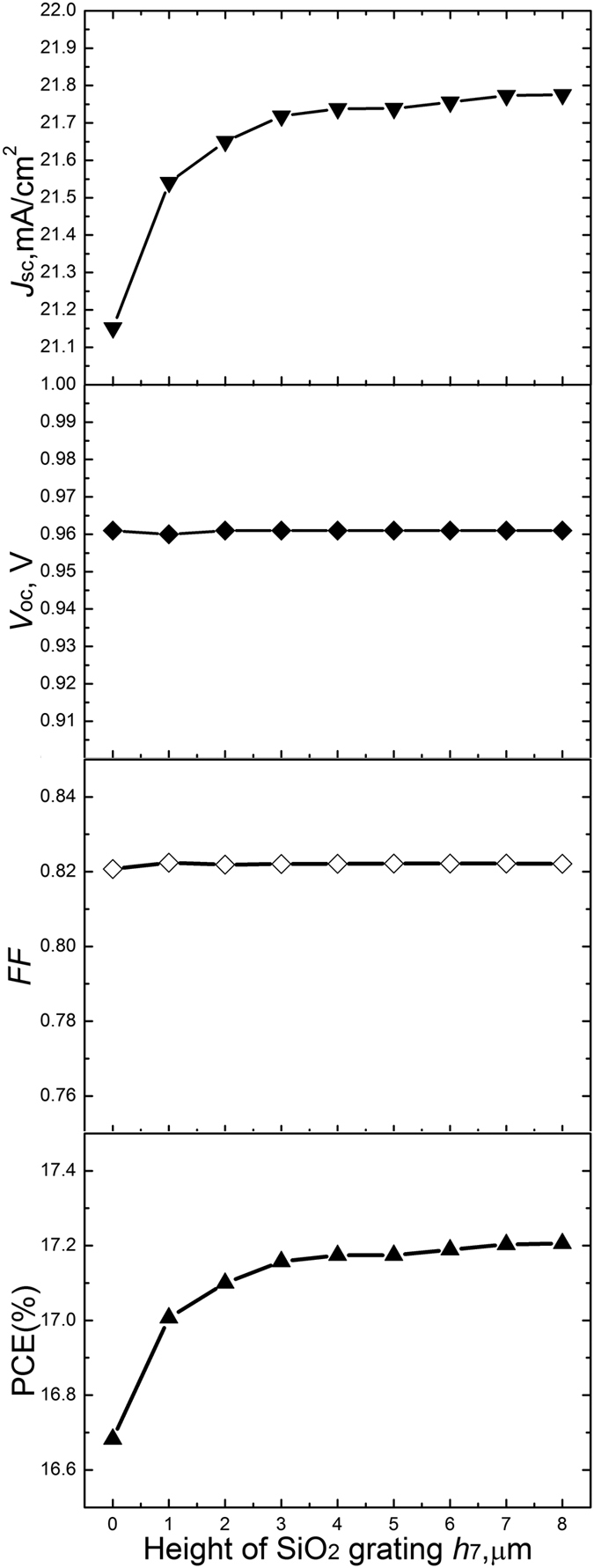
Effect of the SiO_2_ grating height on the electrical properties of structured surface E. The short-circuit currents, open-circuit voltages, fill factors and power conversion efficiencies under AM1.5 one sun illumination of the structured surfaces with different SiO_2_ grating heights are respectively depicted in four illustrations.

For a solar cell employing the moth-eye antireflection structure, the gradient of effective refractive index is reduced so that the reflection is greatly suppressed and the absorption is enhanced with the increase of moth-eye grating height. As the moth-eye grating height is increased furthermore, the absorption tends to a saturation value (Supplementary Fig. S2). Consequently, the electrical properties of the structured surface are also influenced by the height of moth-eye grating. The *J*_sc_, *V*_oc_, *FF* and PCE are four important parameters to evaluate the electrical performance of the solar cell. The effects of the SiO_2_ grating height on these four parameters are shown in Fig. 4. The SiO_2_ grating height of structured surface E is increased from 0 μm to 8 μm by the step of 1 μm. It is seen that the *J*_sc_ is greatly increased as the SiO_2_ grating height is increased from 0 μm to 6 μm. When the SiO_2_ grating height is increased furthermore, the increase of *J*_sc_ is very limited and *J*_sc_ tends to a saturation current of 21.78 mA/cm^2^. The *J*_sc_ is mainly influenced by the intrinsic absorption. Thus the change of *J*_sc_ with the increase of SiO_2_ grating height coincides with the change of intrinsic absorption (Supplementary Fig. S2). *V*_oc_ is almost constant around 0.96 V. This is lower than normal experimental value of 1 V due to significant SRH recombination caused by that the thickness of CH_3_NH_3_PbI_3_ layer is larger than that in the normal experiment. *FF* is almost around 0.82. The PCE becomes higher as the SiO_2_ grating height is increased and finally reaches a saturation value about 17.2%. It has been well known that the PCE of solar cell can be expressed as 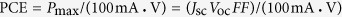, where *P*_max_ is the maximum output power^40^. The *V*_oc_ and *FF* are almost constants. The PCE is mainly influenced by *J*_sc_. Therefore, the change of PCE with the increase of SiO_2_ grating height is the same as that of *J*_sc_. When the SiO_2_ grating height equals 6 μm, the PCE has nearly reaches the saturation value. Thus the SiO_2_ grating height of 6 μm is high enough to improve the performance of perovskite cell and this height is employed in the following sections.

### Effect of the TiO_2_ grating height *h*_8_

**Figure 5:**
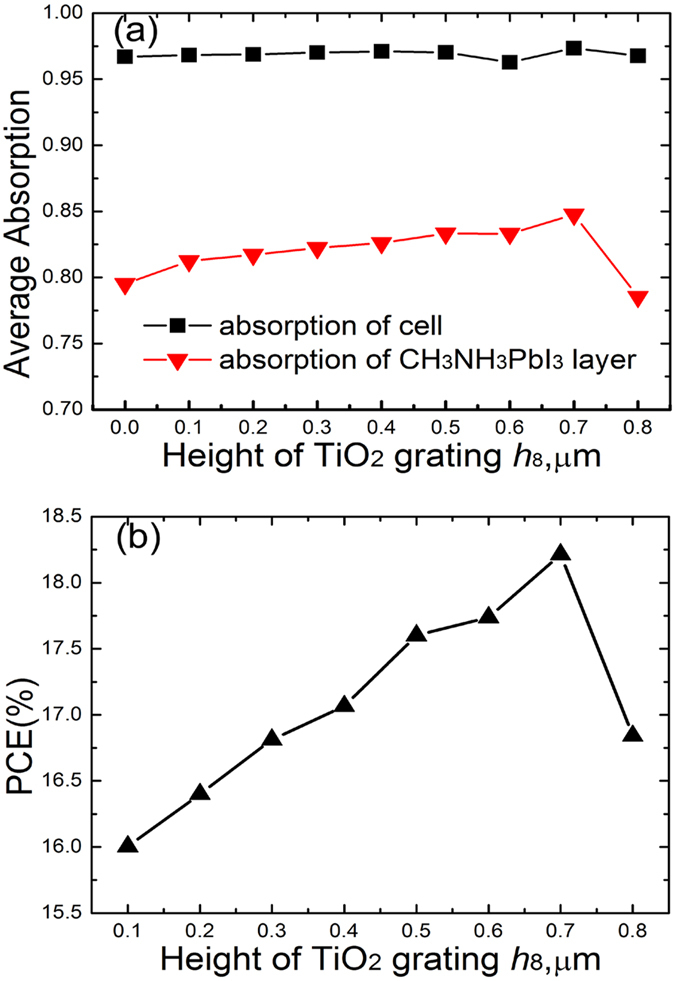
Effect of the TiO_2_ grating height on the optical and electrical performances of the structured surface E. (**a**) The average overall absorption and average intrinsic absorption of the structured surface E with different TiO_2_ grating heights. (**b**) The power conversion efficiencies of the structured surface E with different TiO_2_ grating heights.

Figure 5a shows the effect of the TiO_2_ grating height on the optical performance of structured surface. The FDTD calculated results are integrated with AM 1.5 solar spectrum to acquire the average absorption. It can be seen that the average overall absorption almost keeps constant around 0.97 with the increase of TiO_2_ grating height. The average intrinsic absorption is increased at the beginning with the increase of the TiO_2_ grating height. When the TiO_2_ grating height is increased to 0.7 μm, the average intrinsic absorption reaches the maximum of 0.848 and then is reduced as the TiO_2_ grating height is increased furthermore. Figure 5b shows the effect of TiO_2_ grating height on the PCE of the structured surface E. The PCE is also increased at the beginning with the increase of TiO_2_ grating height. When the TiO_2_ grating height is increased to 0.7 μm, the highest PCE of 18.2% is realized. With the further increase of TiO_2_ grating height, the PCE is reduced. This is attributed to that the intrinsic absorption directly influences the *J*_sc_ of the structured surface. The *J*_sc_ is increased from 20.35 mA/cm^2^ to 22.98 mA/cm^2^ when the TiO_2_ grating height is increased from 0.1 μm to 0.7 μm. As the TiO_2_ grating height is increased from 0.7 μm to 0.8 μm, the *J*_sc_ is reduced from 22.98 mA/cm^2^ to 21.31 mA/cm^2^. This is mainly resulted from that the average intrinsic absorption of structured surface is reduced to 0.848 to 0.785, which leads to the decrease of photon-induced carriers. The *V*_oc_ and the *FF* are almost constants (Supplementary Fig. S3). Therefore, the best size of TiO_2_ grating height is 0.7 μm and this height is employed in the following sections.

### Incident angle dependence and polarization-sensitivity

**Figure 6:**
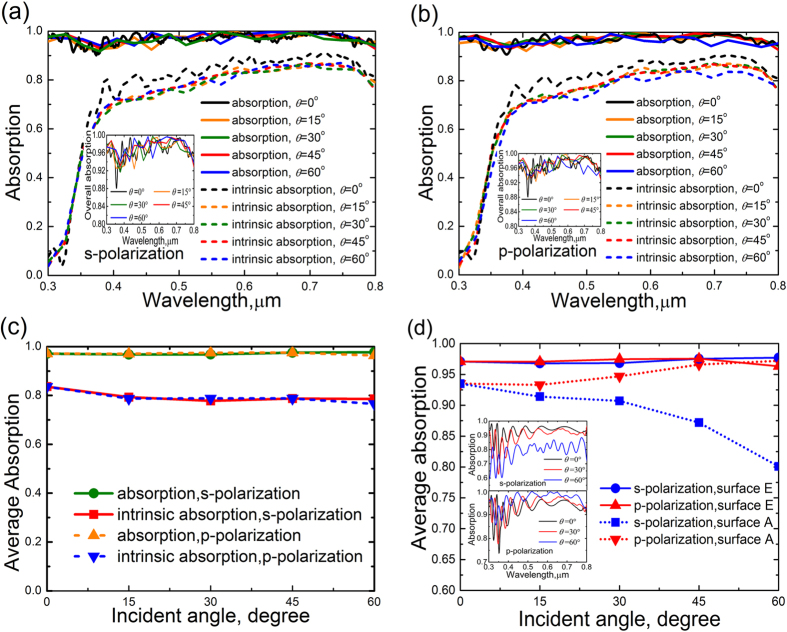
Incident angle-dependence of the spectral absorption for s-polarization and p-polarization of the structured surface E, in which the SiO_2_ moeh-eye grating is combined with TiO_2_ grating. (**a**) S-polarization. The inset shows the enlarged view of overall absorption of at different incident angles for s-polarization. (**b**) P-polarization. The inset shows the enlarged view of overall absorption of at different incident angles for s-polarization. (**c**) Average absorption for s-polarization and p-polarization. (**d**) Comparison between the average absorption of structured surface E and structured surface A at different incident angles for s- and p-polarizations. The inset shows the spectral absorption of structured surface A for s- and p-polarizations at different incident angles.

The omnidirectional characteristics of the micro-structured perovskite solar cell are illustrated by the optical absorption for various incident angle varying from 0° to 60° by the step of 15°, as shown in Fig. 6. Since the polarization of the incident wave strongly influences the spectral features of the structured surface at oblique incidence, both the s- and p-polarizations are considered. Figure 6a shows the absorption of the structured surface at different incident angles for s-polarization. Both the overall absorption and intrinsic absorption exhibit perfect omnidirectional spectral features for incident angle ranging from 0° to 60°. The inset in Fig. 6a shows the much clearer and enlarged view of the overall absorption at different incident angles for s-polarization. The overall absorption spectra slightly oscillate around 0.96 and the incident angle has little effect on the high absorption of the structured surface E. The intrinsic absorption at oblique incidence is slightly lower than that at the incident angle of 0°. However, the intrinsic absorption still keeps higher than 0.7 at the wavelength from 0.4 μm to 0.8 μm at oblique incidence, even the incident angle is increased to as large as 60°. This states that the structured surface of perovskite cell possesses the omnidirectional features for s-polarization. Figure 6b depicts the absorption of the structured surface E at different incident angles for p-polarization. The inset in Fig. 6b is the shows the enlarged view of the overall absorption at different incident angles for p-polarization. The effect of incident angle on the overall absorption is negligible and the overall absorption in the wavelength band of 0.3–0.8 μm keeps a high level around 0.96. The intrinsic absorption is slightly reduced with the increase of incident angle while the intrinsic absorption is always above 0.7 at the wavelength from 0.4 μm to 0.8 μm. It is demonstrated that the omnidirectional feature is also realized by the structured surface E for p-polarization. In order to evaluate the overall optical omnidirectional characteristic of the structured surface E, the average overall absorption and the average intrinsic absorption are illustrated in Fig. 6c. The average absorption of the structured surface E for s-polarization almost coincides with that for p-polarization. The structured surface maintains a high overall absorption around 0.97 in the wide incident angle of 0°–60°. The average intrinsic absorption is slightly reduced with the increase of incident angle. However, the reduction is very limited and the intrinsic absorption is maintained at a high level. As the incident angle is increased from 0° to 60°, the average intrinsic absorption is reduced from 0.836 to 0.786 for s-polarization and from 0.836 to 0.766 for p-polarization. The high intrinsic absorption is effectively realized in a wide incident angle range of 0°–60°. In order to highlight the advantage of the structured surface E on light trapping, the average absorption of structured surface E at different incident angles for s- and p-polarizations is compared with that of structured surface A (planar film in Fig. 2a) in Fig. 6d. The inset in Fig. 6d shows the spectral absorption of structured surface A. The absorption of structured surface A is obviously polarization-sensitive and incident angle-dependent. In contrast, the variation of the average absorption of structured surface E for both the s- and p-polarizations is negligible. Simultaneously, the absorption of structured surface E for both the s- and p-polarizations is remarkably larger than that of structured surface A in a wide incident angle range. Therefore, the structured surface E exhibits great superiority of omnidirectional and polarization-insensitive light-trapping compared with the structured surface A.

## Discussion

In conclusion, we have developed a comprehensive design approach for omnidirectional high-performance perovskite solar cell with coupled optical-electrical simulations, in which the selection of structured surface, effect of geometry parameters, incident-angle dependence and polarization-sensitivity are considered. The optical and electrical performances of five different structured surfaces are theoretically investigated. The simulated results are compared to select a best structured sruface for perovskite cell. The results reveal that the CH_3_NH_3_PbI_3_ grating can obviously enhance the absorption of CH_3_NH_3_PbI_3_ layer to generate more electron-hole pairs while the recombination is greatly increased and the *J*_sc_ is greatly reduced for the significant surface recombination velocity. The structured surface with TiO_2_ grating and SiO_2_ grating is the best structured surface for its high absorption due to the gradient refractive index of SiO_2_ grating and double scatterings of TiO_2_ grating and SiO_2_ grating. Simultaneously, the TiO_2_ grating and SiO_2_ grating have little effect on the recombination of perovskite cell and the efficiency of the cell is greatly increased. The absorption is enhanced with the increase of SiO_2_ grating height due to the reduced gradient of effective refractive index. The TiO_2_ grating can effectively enhance the intrinsic absorption of perovskite solar cell due to the scattering of TiO_2_ grating and the TiO_2_ grating height of 0.7 μm shows the best absorption. The power conversion efficiency can be increased to 18.2% with *J*_sc_ = 22.98 mA/cm^2^ and *V*_oc_ = 0.96 V. The structured surface E exhibits excellent omnidirectional high absorption in the wide incident angle range of 0°–60° and the structured surface possesses favorable polarization-insensitive features. As such, the comprehensive design approach investigated in this paper can highlight the better utilizing of the solar energy with perovskite cell.

## Methods

The evaluation of PV cell performance contains two aspects: one is the optical absorption and the other is carrier transport. The optical absorption can be calculated by FDTD method, which is developed for solving Maxwells’ equations that describes the light propagation and interactions with the cell surface^41^


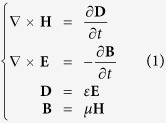


where **E** is the electric field, **H** is the magnetic field, **D** is the electric displacement field, **B** is the magnetic flux density, *ε* is the complex permittivity, and *μ* is the complex permeability. Since *ε* is frequency-dependent, the frequency domain equation 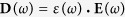 should be transformed into the time domain by the auxiliary difference equation (ADE). Convergence was assured by using a time step determined by^41^


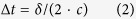


where *δ* is the smallest spatial increment and *c* is the speed of light in vacuum. The mesh size significantly influences the convergence and accuracy of the simulation, as reported by Barnes^42^. The mesh size and the time step are respectively set to 2 nm and 0.002 fs to ensure the convergence and accuracy of the calculation at the normal incidence. Since the structured surface is periodic in x- and y- directions, the periodic boundary conditions are applied in x- and y- directions at normal incidence meanwhile Bloch boundary conditions are applied for oblique incidence. The perfectly matched layers (PML) are used in z-direction. FDTD is a mature numerical method which is extensively used in computational electromagnetics. The detailed algorithm of FDTD method has been widely reported and one can find it in many literatures^43^. In order to evaluate the overall optical performance of the perovskite-based solar cells, the average absorption is calculated by^44^


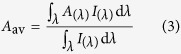


where *A*_(*λ*)_ is the spectral absorption. *I*_(*λ*)_ is for AM 1.5 emissions^45^.

The numerical simulation at large incident angles become difficult on some extent. Oh and Escuti developed an efficient implementation of FDTD method through periodic media with arbitrary anisotropy and the FDTD algorithm is validated against rigorous coupled-wave analysis method at oblique incidence^46^. In order to ensure the accuracy and acceptable convergence of the FDTD simulation at larger oblique incidence, the minimum mesh size of 1 nm, the modified PMLs with 256 layers and the Bloch boundary condition are used in the calculation. The FDTD algorithm in this paper is validated to be reliable at large incident angle by the agreement between the FDTD simulated results of film stack at incident angle of 45° and 60° and the results calculated by the well-known transfer-matrix formalism (Supplementary Fig. S4). The average values of spectral absorption calculated by FDTD and transfer-matrix methods are compared (Supplementary Table S2) to evaluate the overall simulation accuracy. The negligible difference between the average values of absorption calculated by FDTD and transfer-matrix methods proves that simulated results by FDTD can reveal the absorption performance of the structured surface at oblique incidence, even at large incident angles.

By assuming that each photon absorbed by CH_3_NH_3_PbI_3_ layer can generate an electron-hole pair, the optical generation can be expressed as^47^


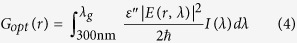


where *E* is the electric field, *ε*″ is the imaginary part of the permittivity of the semiconductor material, 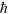 is the reduced Planck constant, and *λ*_*g*_ is the cut-off wavelength corresponding to the band gap of the semiconductor. The carrier transport process can be described by the coupled nonlinear equations of Poisson, continuity, and drift-diffusion equations as^48^,^49^,^50^


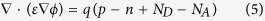



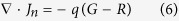



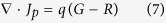



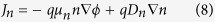



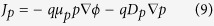


where *q* stands for the electron charge, *ϕ* for the electrical potential, *n*(*p*) for the electron (hole) concentration, *N*_*D*_(*N*_*A*_) for the donor (acceptor) doping concentration, *J*_*n*_(*J*_*p*_) for the current density of electron (hole), *G* for the optical generation rate obtained from optical simulation, *R* is the carrier recombination rate, *μ*_*n*_(*μ*_*p*_) is the electron (hole) mobility, and *D*_*n*_(*D*_*p*_) is the electron (hole) diffusion coefficient. The Shockley-Read-Hall recombination and surface recombination are involved in the electrical simulation as^48^


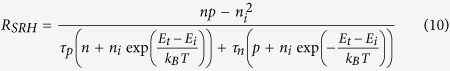



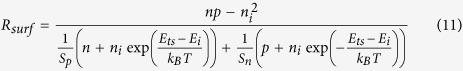


where *τ*_*n*_(*τ*_*p*_) for the electron (hole) lifetime, *n*_*i*_ for the intrinsic carrier concentration, and *S*_*n*_(*S*_*p*_) is the surface recombination velocity of electron (hole). *E*_*t*_ is the defect energy level. *E*_*i*_ is the Femi energy level. *E*_*ts*_ is the surface defect energy level. *k*_*B*_ is the Boltzmann constant.

For the semiconductor material, the electron (hole) lifetime is related to effective electron (hole) mass. The effective electron (hole) mass can be expressed as^51^


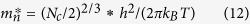



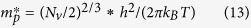


where, *N*_*c*_ and *N*_*v*_ are respectively the effectively conduction band density and valence band density. *h* is the Planck constant. *T* is the cell temperature and *T* = 300 K is selected for the electrical simulation. Consequently, the electron (hole) lifetime are^52^,^53^


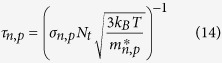


where, *σ*_*n*,*p*_ is the capture cross-section. *N*_*t*_ is the defect density.

In a semiconductor or conductor, the work function describes the energy cost of removing an electron from the intrinsic energy level and placing is at infinity. This property directly influences the charge output. The workfunction of the semiconductor can be expressed as^54^


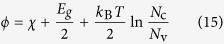


where *χ* is the electron affinity and *E*_*g*_ is the band gap energy.

## Additional Information

**How to cite this article**: Zhang, Y. and Xuan, Y. Comprehensive design of omnidirectional high-performance perovskite solar cells. *Sci. Rep.* **6**, 29705; doi: 10.1038/srep29705 (2016).

## Supplementary Material

Supplementary Information

## Figures and Tables

**Table 1:** Geometry parameters of five structured surfaces of perovskite solar cells.

Structure	*h*_1_(μm)	*h*_2_(μm)	*h*_3_(μm)	*h*_4_(μm)	*h*_5_(μm)	*h*_6_(μm)	*h*_7_(μm)	*h*_8_(μm)	*h*_9_(μm)
Structured surface A	0.01	0.35	0.75	0.05	1	20	—	—	—
Structured surface B	0.01	0.35	0.75	0.05	1	20	—	0.4	0.4
Structured surface C	0.01	0.35	0.75	0.05	1	20	—	0.4	—
Structured surface D	0.01	0.35	0.75	0.05	1	20	6	—	—
Structured surface E	0.01	0.35	0.75	0.05	1	20	6	0.4	—

## References

[b1] Cheng Y. J., Yang S. H. & Hsu Synthesis of conjugated polymers for organic solar cell applications. Chem. Rev. 109, 5868–5923 (2009). 19785455 10.1021/cr900182s

[b2] Adachi M. M., Anantram M. P. & Karim K. S. Core-shell silicon nanowire solar cells. Sci. Rep. 3, 1–6 (2013). 10.1038/srep01546 PMC3607835 23529071

[b3] Liu Y. . Solution-processed small-molecule solar cells: breaking the 10% power conversion efficiency. Sci. Rep. 3, 1–8 (2013). 10.1038/srep03356 PMC3842540 24285006

[b4] Holman Z. C. . Parasitic absorption in the rear reflector of a silicon solar cell: simulation and measurement of a sub-bandgap reflectance for common dielectric/emtal relfectors. Sol. Energ. Mat. Sol. C. 120, 426–430 (2014).

[b5] Makableh Y. F. . Enhancement of GaAs solar cell performance by using a ZnO sol-gel anti-reflection coating. Sol. Energ. Mat. Sol. C. 123, 178–182 (2014).

[b6] Ren Z. . Thermal assisted oxygen annealing for high efficiency planar CH_3_NH_3_PbI_3_ perovskite solar cell. Sci. Rep. 4, 6752 (2014). 25341527 10.1038/srep06752 PMC4208060

[b7] Noel N. K. . Lead-free organic-inorganic tin halide perovskite for photovoltaic applications. Energy Environ. Sci. 7, 0.61-3068 (2014).

[b8] Tress W. . Understanding the rate-dependent J-V hysteresis, slow time component, and aging in CH3NH3PbI3 perovskite solar cells: the role of a compensated electric field. Energy Environ. Sci. 8, 995–1004 (2015).

[b9] Wang K. C. . P-type mesoscopic nickel oxide/organometallic perovskite heterojunction solar cells. Sci. Rep. 4, 4756 (2014). 24755642 10.1038/srep04756 PMC3996464

[b10] Brivio F., Walker A. B. & Walsh A. Structural and electronic properties of hybrid perovskite for high-efficiency thin-film photovoltaics from first-principles. Apl. Mater. 1, 042111 (2013).

[b11] Baikie T. . Synthesis and crystal chemistry of the hybrid perovskite (CH_3_NH_3_)PbI_3_ for solid-state sensitized solar cell applications. J. Mater. Chem. A 1, 5628 (2013).

[b12] Okamoto Y. & Suzuki Y. Perovskite-type SrTiO3, CaTiO3 and BaTiO3 porous film electrodes for dye-sensitized solar cells. J. Ceram. Soc. Jpn. 122, 728–731 (2014).

[b13] Eperon G. E., Burlakov V. M., Docampo P., Goriely A. & Snaith H. J. Morphological control for high performance, solution-processed planar heterojunction perovskite solar cells. Adv. Funct. Mater. 24, 151–157 (2014).

[b14] Umari P., Mosconi E. & Angelis F. D. Relativistic GW calculations on CH_3_NH_3_PbI_3_ and CH_3_NH_3_SnI_3_ perovskite for solar cell applications. Sci. Rep. 4, 4467 (2014). 24667758 10.1038/srep04467 PMC5394751

[b15] Snaith H. J. . Efficiency enhancements in solid-state hybrid solar cells via reduced charge recombination and increased light capture. Nano Lett. 7, 3372–3376 (2007). 17918905 10.1021/nl071656u

[b16] Bach U. . Solid-state dye-sensitized mesoporous TiO2 solar cells with high photon-to-electron conversion efficiencies. Nature 395, 583–585 (1998).

[b17] Kim H. S. . Lead lodide perovskite sensitized all-solid-state submicron thin film mesoscopic solar cell with efficiency exceeding 9%. Sci. Rep. 2, 1–7 (2012). 10.1038/srep00591 PMC3423636 22912919

[b18] Lee M. M., Teuscher J., Miyasaka T., Murakami T. N. & Snaith H. J. Efficient hybrid solar cells based on meso-superstructured organometal halide perovskites. Science 338, 643–647 (2012). 23042296 10.1126/science.1228604

[b19] Kim H. S. . High efficiency solid-state sensitized solar cell-based on submicrometer butile TiO_2_ nanorod and CH_3_NH_3_PbI_3_ perovskite sensitizer. Nano Lett. 13, 2412–2417 (2013). 23672481 10.1021/nl400286w

[b20] Stranks S. D. . Electron-hole diffusion lengths exceeding 1 micrometer in an organometal trihalide perovskite absorber. Science 342, 341–344 (2013). 24136964 10.1126/science.1243982

[b21] Ball J. M., Lee M. M., Hey A. & Snaith H. J. Low-temperature processed meso-superstructured to thin-film perovskite solar cells. Energy Environ. Sci. 6, 1739–1743 (2013).

[b22] Liu M., Johnston M. B. & Snaith H. J. Efficient planar heterojunction perovskite solar cells by vapour deposition. Nature 501, 395–398 (2013). 24025775 10.1038/nature12509

[b23] Snaith H. J. Perovskite: the emergence of a new era for low-cost, high-efficiency solar cells. J. Phys. Chem. Lett. 4, 3623–3630 (2013).

[b24] Liu D. & Kelly T. L. Perovskite solar cells with a planar heterojunction structure prepared using room-temperature solution processing techniques. Nature Photon. 8, 133–138 (2014).

[b25] Zhou H. . Interface engineering of highly efficient perovskite solar cells. Science 345, 542–546 (2014). 25082698 10.1126/science.1254050

[b26] NREL, *Best research-cell efficiencies*. (2015) Available at: http://www.nrel.gov/ncpv/images/efficiency_chart.jpg. (Accessed: 18th November 2015).

[b27] Liu F. . Numerical simulation: toward the design of the high-efficiency planar perovskite solar cells. Appl. Phys. Lett. 104, 253508 (2014).

[b28] Ball J. M. . Optical properties and limiting photocurrent of thin-film perovskite solar cells. Energy Environ. Sci. 8, 602–609 (2015).

[b29] Spinelli P., Verschuuren M. A. & Polman A. Broadband omnidirectional antireflection coating based on subwavelength surface Mie resonators. Nat. Commun. 3, 1–5 (2012). 10.1038/ncomms1691 PMC3338005 22353722

[b30] Song Y. M., Jang S. J., Yu J. S. & Lee Y. T. Bioinspired parabola subwavelength structures for improved broadband antireflection. Small 6, 984–987 (2010). 20461734 10.1002/smll.201000079

[b31] Tommila A. . Moth-eye antireflection coating fabricated by nanoimprint lighography on 1 eV dilute nitride solar cell. Prog. Photovolt. Res. Appl. 21, 1158–1162 (2013).

[b32] Yamada N., Ijiro T., Okamoto E., Hayashi K. & Masuda H. Characterization of antireflection moth-eye film on crystalline silicon photovoltaic module. Opt. Express 19, A118–A125 (2011). 21445213 10.1364/OE.19.00A118

[b33] Han L. . GaN microdomes for broadband omnidirectional antireflection for concentrator photovoltaics. *In SPIE OPTO* 862016 (International Society for Optics and Photonics, 2013).

[b34] Ball J. M. Supplementary information for “optical properties and limiting photocurrent of thin-film perovskite solar cells”. Energy Environ. Sci. 8 (2015).

[b35] Palik E. D. Handbook of Optical Constants of Solids (Academic press, New York, 1985).

[b36] Raut H. K., Ganesh V. A., Nair A. S. & Ramakrishna S. Anti-reflective coatings: a critical, in-depth review. Energy Environ. Sci. 4, 3779–3804 (2011).

[b37] Balenzategui J. L. & Chenlo F. Measurement and analysis of angular response of bare and encapsulated silicon solar cells. Sol. Energ. Mat. Sol. C. 86, 53–83 (2005).

[b38] Wang F. . Development of nanopatterned fluorine-doped tin oxide electrodes for dye-sensitized solar cells with improved light trapping. Appl. Mater. Interfaces 4, 1565–1572 (2012). 10.1021/am201760q 22324513

[b39] Lee J., Park J. & Lee M. Nanostructured TiO2 diffraction grating fabricated via imprinting and TiCl_4_ treatment. J. Mater. Chem. C 2, 981–985 (2014).

[b40] Park K. T. . Lossless hybridization between photovoltaic and thermoelectric devices. Sci. Rep. 3, 1–6 (2013). 10.1038/srep02123 PMC3699810 23820973

[b41] Yang L. L., Xuan Y. M., Han Y. G. & Tan J. J. Investigation on the performance enhancement of silicon solar cells with an assembly grating structure. Energy Convers. Manage 54, 30–37 (2012).

[b42] Barnes W. L. Comparing experiment and theory in plasmonics. J. Opt. A: Pure Appl. Opt. 11, 114002 (2009).

[b43] Ge D. & Yan Y. Finite-Difference Time-Domain Method for Electromagnetic Waves (Publishing house of Xidian University, Xi’an, 2011).

[b44] Meng X. . Combined front and back diffraction gratings for broad band light trapping in thin film solar cell. Opt. Express 20, A560–A571 (2012). 23037523 10.1364/OE.20.00A560

[b45] Website for NREL’s AM1.5 Standard Dataset. *Reference solar spectral irradiance: air mass 1.5.* (2015). Available at: http://rredc.nrel.gov/solar/spectra/am1.5. (Accessed: 18th November 2015).

[b46] Oh C. & Escuti M. J. Time-domain analysis of periodic anisotropic media at oblique incidence: an efficient FDTD implementation. Opt. Express 14, 11870–11884 (2006). 19529610 10.1364/oe.14.011870

[b47] Da Y. & Xuan Y. M. Role of surface recombination in affecting the efficiency of nanostrucutred thin-film solar cells. Opt. Express 21, A1065–A1077 (2013). 24514926 10.1364/OE.21.0A1065

[b48] Selberherr S. Analysis and Simulation of Semiconductor Device (Springer Science & Business Media, New York, 2012).

[b49] Sha W. E., Choy W. C., Wu Y. & Chew W. C. Optical and electrical study of organic solar cells with a 2D grating anode. Opt. Express 20, 2572–2580 (2012). 22330495 10.1364/OE.20.002572

[b50] Li X. . Multi-dimensional modeling of solar cells with electromagnetic and carrier transport calculations. Prog. Photovolt. Res. Appl. 21, 109–120 (2013).

[b51] Zhao Y. Q., Yao S. Y. & Shi Z. F. Semiconductor physics and devices basic principles (Publishing house of electronics industry, Beijing, 2013).

[b52] Schroder D. K. Carrier lifetimes in Silicon. IEEE Transactions on Electron Devices 44, 160–170 (1997)

[b53] Henry C. H. & Lang D. V. Nonradiative capture and recombination by multiphonon emission in GaAs and GaP. Phys. Rev. B 15, 989–1016 (1977).

[b54] Anderson B. L. & Anderson R. L. Fundamentals of Semiconductor Devices (McGraw-Hill, Inc.: New York,, 2004).

