# Supplementary material for: Comprehensive design of omnidirectional high-performance perovskite solar cells
Source: Sci Rep. 2016 Jul 13;6:29705. doi: 10.1038/srep29705 (PMC4942834; doi:10.1038/srep29705)
Supplement: Supplementary Information [file srep29705-s1.doc]

**Supplementary Information**

**Comprehensive design of omnidirectional high-performance perovskite solar cells**

Yutao Zhang^1^, Yimin Xuan1,2*[[1]](#footnote-2) 

^1^School of Energy and Power Engineering, Nanjing University of Science& Technology, Nanjing 210094, China，

^2^School of Energy and Power Engineering, Nanjing University of Aeronautics &Astronautics, Nanjing 210094, China.

Table S1 The electrical parameters of the materials in perovskite solar cells

| Parameters | FTO | TiO_2_ | CH_3_NH_3_PbI_3_ | HTM | Ag |
| --- | --- | --- | --- | --- | --- |
| *N*_A_ (cm^-3^) | — | — | — | 2×10^18a^ | — |
| *N*_D_ (cm^-3^) | — | 10^16b^ | 10^13b^ | — | — |
| *μ*_n_/* μ*_p_(cm^2^/V s) | — | 20/10^b^ | 50/50^c^ | 0.0002/0.0002^d^ | — |
| *E*_g_ (eV) | — | 3.2^b^ | 1.55^e^ | 3.0^d^ | — |
| *χ* (Ev) | — | 3.9^b^ | 3.9^f^ | 2.45^d^ | — |
| *N*_t_ (cm^-3^) | — | 1×10^15b^ | 2.5×10^13b^ | 1×10^15b^ | — |
| *Φ* (eV) | 4.4^c^ | 5.53 | 4.702 | 3.98 | 4.26^g^ |
| *ε* | 3.5^h^ | 9^b^ | 6.5^b^ | 3^b^ | -10^i^ |

^a^Data were taken from Ref. 1; ^b^Data were taken from Ref. 2; ^c^Data were taken from Ref. 3;

^d^Data were taken from Ref. 4; ^e^Data were taken from Ref. 5; ^f^Data were taken from Ref. 6;

^g^Data were taken from Ref. 7; ^h^Data were taken from Ref. 8; ^i^Data were taken from Ref. 9;

The electrical parameters not mentioned in Table 1 are set to be identical in all layers of perovskite solar cell. The effect densities of conductor band and valence band are *N*_c_=1.8×10^19^ and *N*_v_=2.2×10^18^ cm^-3^. The capture cross-section of electron and hole is *σ*=2×10^-14^ cm^-2^. The thermal velocity of electron and hole is 10^7^ cm/s^2^. The surface recombineation velocity is 20000 cm/s.


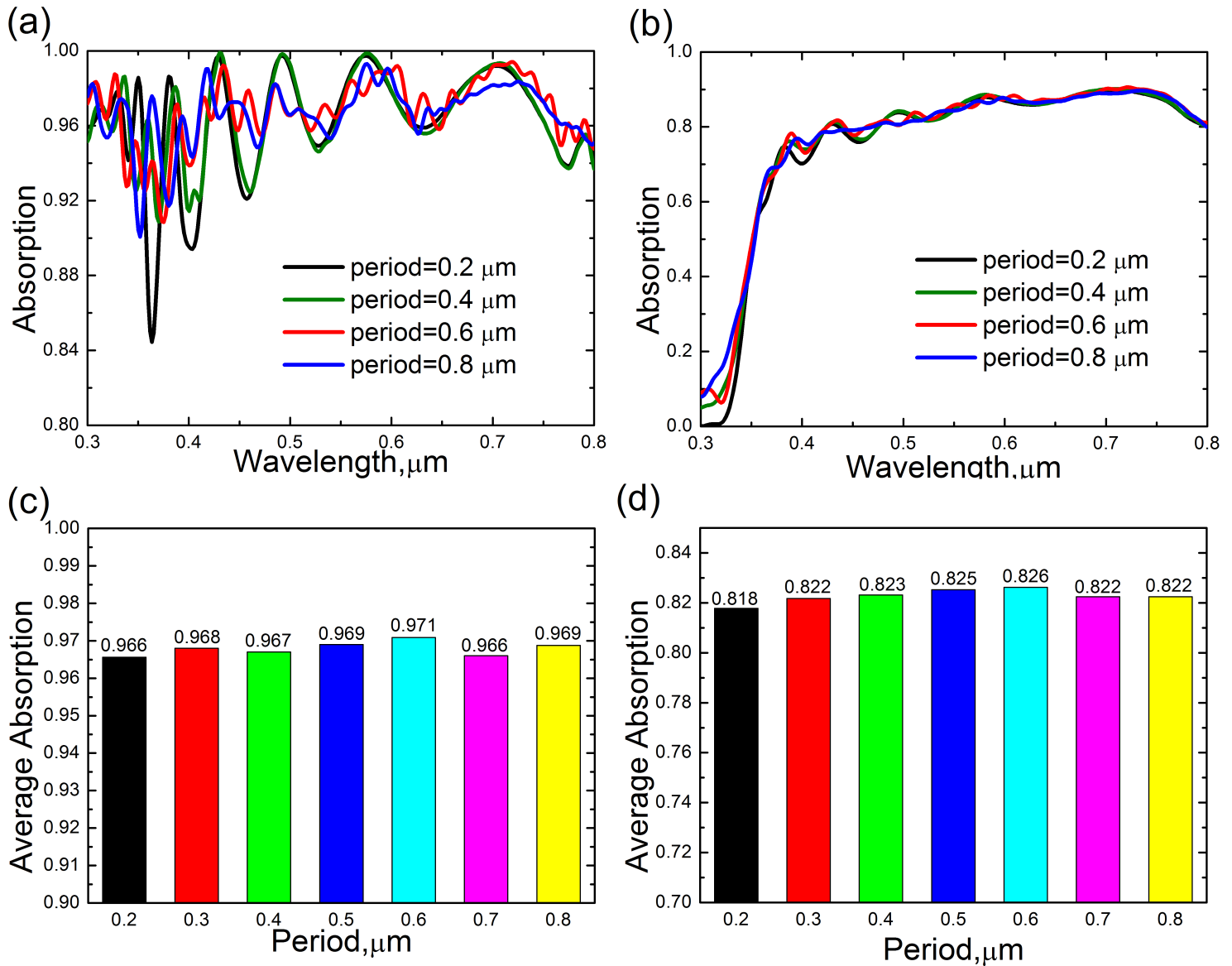


igure S1| Effect of the period on the spectral absorption properties of structured surface with TiO_2_ grating and SiO_2_ grating. The period is increased from 0.2 μm to 0.8 μm with the step of 0.1 μm. (a) Overall absorption with different periods. (b) Intrinsic absorption with different periods. (c) Average overall absorption with different periods. (d) Average intrinsic absorption with different periods.


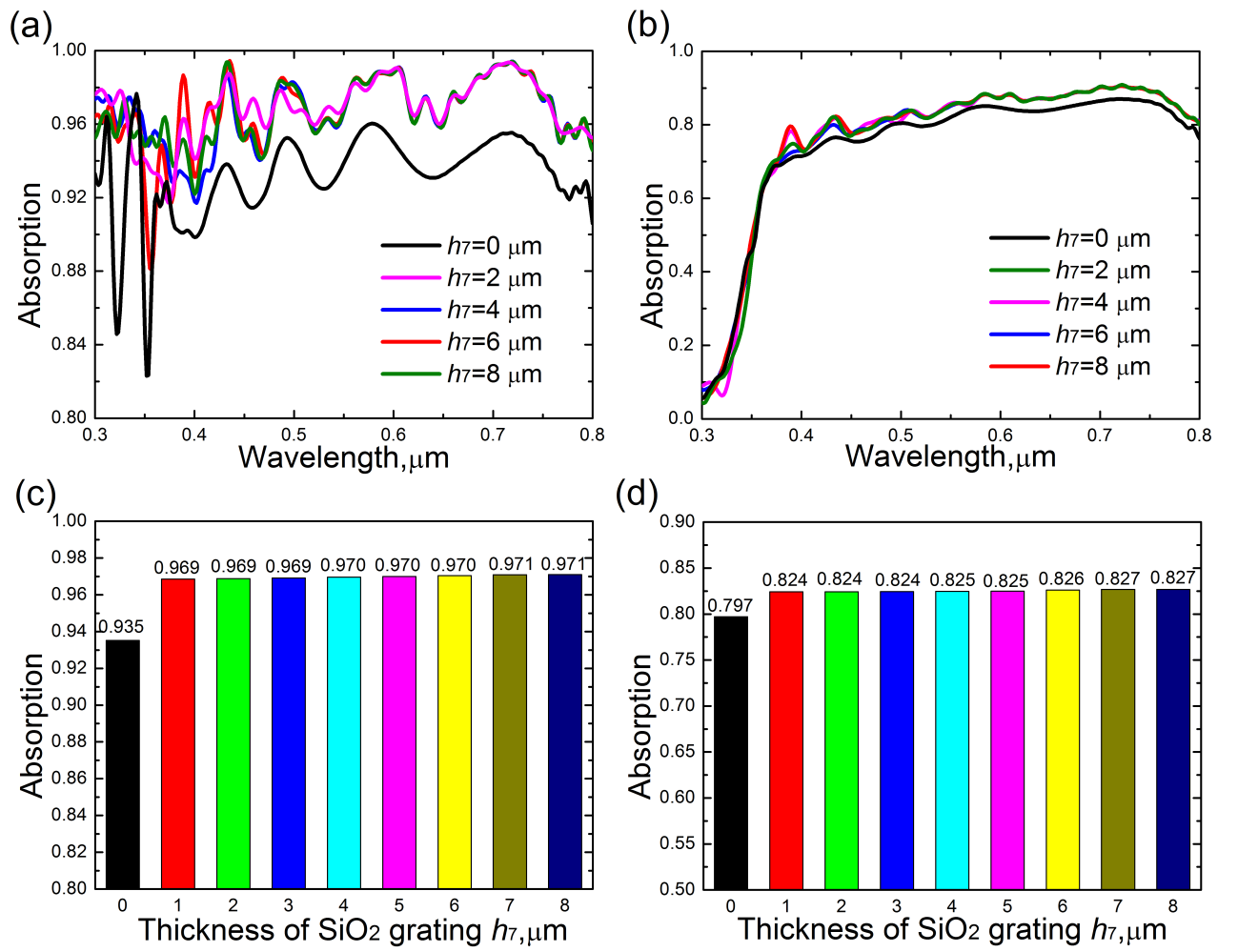


Figure S2 | Effect of the SiO_2_ grating height on the spectral absorption properties of structured surface with TiO_2_ grating and SiO_2_ grating. The SiO_2_ grating height is increased from 0 μm to 8 μm with the step of 1 μm. (a) Overall absorption with different SiO_2_ grating heights. (b) Intrinsic absorption with different SiO_2_ grating heights. (c) Average overall absorption with different SiO_2_ grating heights. (d) Average intrinsic absorption with different SiO_2_ grating heights.


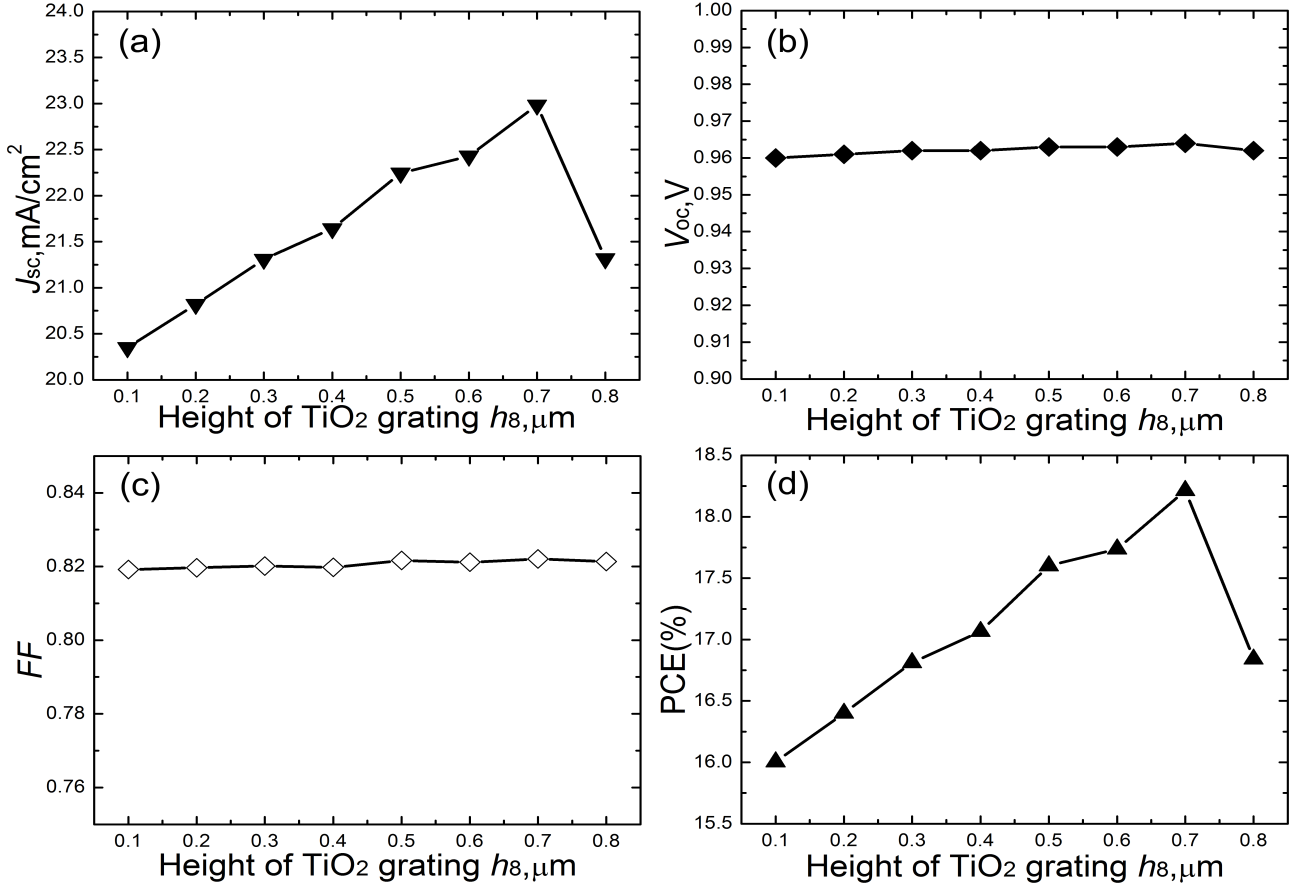


Figure S3 | Effect of TiO_2_ grating height on the electrical performances of the structured surfaces with TiO_2_ grating and SiO_2_ grating. (a) Short-circuit currents with different TiO_2_ grating heights. (b) Open-circuit voltages with different TiO_2_ grating heights. (c) Fill factors with different TiO_2_ grating heights. (d) Power conversion efficiencies with different TiO_2_ grating heights.


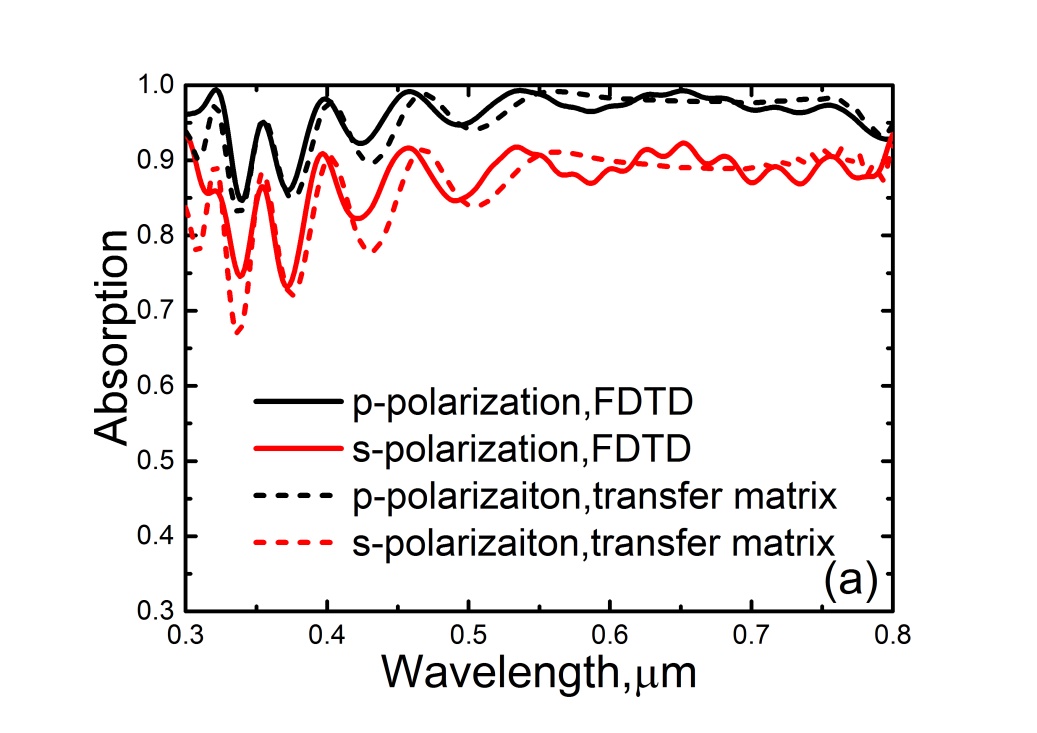

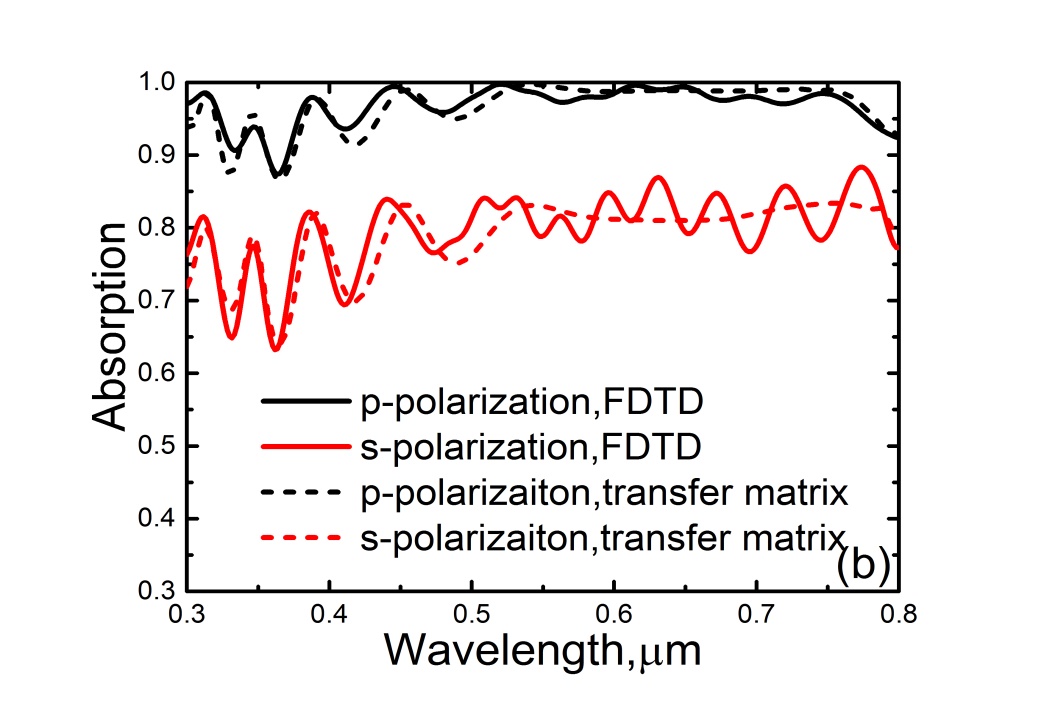


Figure S4 | Comparison between FDTD simulation and transfer matrix simulation of the absorption spectra at the incident angle of 45ºand 60°for s- and p-polarizations of structured surface A, (a) 45º，(b) 60º. The minimum mesh size of 1 nm, 256 PMLs and Bloch boundary condition are employed to ensure the accuracy and good convergence. The FDTD simulated results coincide with the results obtained by transfer matrix method. At the incident angle of 45º, the maximum deviations between the spectral absorption calculated by FDTD and transfer matrix method are 0.080 for s-polarization and 0.031 for p-polarization. At incident angle of 60º, the maximum deviations between the spectral absorption calculated by FDTD and transfer matrix method are 0.058 for s-polarization and 0.036 for p-polarization, respectively. The FDTD algorithm can be confirmed to be reliable at the large incident angle. The somewhat deviation is attributed to the difference between FDTD method and transfer method.

Table S2 The average values of the spectral absorption calculated by transfer matrix and FDTD methods of structured surface A at the incident angle of 45º and 60º

| incident angle | s-polarization | | p-polarization | |
| --- | --- | --- | --- | --- |
|  | transfer matrix | FDTD | transfer matrix | FDTD |
| 45º | 0.876 | 0.881 | 0.964 | 0.966 |
| 60º | 0.799 | 0.805 | 0.973 | 0.973 |

The average values of spectral absorption calculated by FDTD and transfer-matrix methods of the structured surface A at the incident angle of 45º and 60º for s- and p-polarizations are listed in Table S2. The differences between the average values of FDTD and transfer-matrix simulated results at the incident angle of 45º and 60º for s- and p-polarizations are negligible. This proves the FDTD simulations at large incident angle are reliable and can prove the omnidirectional characteristics of micro-structured surface.

**References**

Snaith, H. J. & Gratzel, M. Electron and hole transport through mesoporous TiO2 infiltrated with spiro-MeOTHAD. *Adv. Mater.* **19**, 3643-3647 (2007).

Minemoto, T. & Murata, M. Device modeling of perovskite solar cells based on structural similarity with thin film inorganic semiconductor solar cells. *J. Appl. Phys.* **105**, 083906 (2014).

1. Liu, F. *et al*. Numerical simulation: toward the design of the high-efficiency planar perovskite solar cells. *Appl. Phys. Lett.* **104**, 253508 (2014).
2. Liu, D. & Kelly, T. L. Perovskite solar cells with a planar heterojunction structure prepared using room-temperature solution processing techniques. *Nature Photon.* **8**, 133-138 (2014).
3. Lee, M. M., Teuscher, J., Miyasaka, T., Murakami, T. N. & Snaith, H. J. Efficient hybrid solar cells based on meso-superstructured organometal halide perovskites. *Science* **338**, 643-647 (2012).

Noh, J. H., Im, S. H., Heo, J. H., Mandal, T. N. & Seok, S. I. Chemical management for colorfull, efficient, and stable inorganic-organic hybrid nanostructured solar cells. *Nano Lett.***13**, 1764-1769 (2013).

Michaelson, H. B. The work function of the elements and its periodicity. *J. Appl. Phys.* **48**, 4729 (1977).

1. Ball, J. M. *et al*. Supplementary information for: “Optical properties and limiting photocurrent of thin-film perovskite solar cells”. *Energy Environ. Sci.* **8**, (2015).

Palik, E. D. *Handbook of Optical Constants of Solids* (Academic press, New York, 1985).

1. Corresponding author. Tel.: +86 025 84315700; fax: +86 025 84315991

   E-mail address: ymxuan@mail.njust.edu.cn [↑](#footnote-ref-2)
